# Pemetrexed, Carboplatin, and Concomitant Radiation followed by Surgery for Locally Advanced Esophageal Cancer: Results of a Planned Interim Toxicity Analysis of North Central Cancer Treatment Group Study N044E

**DOI:** 10.4137/cmo.s444

**Published:** 2008-04-01

**Authors:** Rajini Katipamula, Aminah Jatoi, Nathan R. Foster, Francis Nichols, Joseph Rubin, Matthew Callister, Leonard Gunderson, Steven Alberts

**Affiliations:** 1 Department of Oncology, Mayo Clinic, Rochester, Minnesota; 2 Division of Biostatistics, Mayo Clinic, Rochester, Minnesota; 3 Division of Thoracic Surgery, Mayo Clinic, Rochester, Minnesota; 4 Department of Radiation Oncology, Mayo Clinic, Scottsdale, Arizona

## Abstract

**Purpose:**

This brief report describes a planned, interim, 6-patient toxicity analysis that confirms the safety of pemetrexed, carboplatin, radiation with subsequent surgery, as prescribed in the North Central Cancer Treatment Group trial N044E, in patients with locally advanced esophageal cancer.

**Methods:**

Six patients with locally advanced, potentially resectable esophageal cancer received pemetrexed 500 mg/m^2^ and carboplatin AUC = 6 on days 1 and 22 with 5040 centigray of concomitant radiation in 28 fractions over 5.5 weeks followed by esophagectomy as a prelude to a phase II multi-institutional trial.

**Results:**

Only 1 of the 6 patients experienced a grade 4 adverse event (neutropenia). This patient also experienced a grade 3 depression. Of the remaining 5 patients, three experienced at least one grade 3 adverse event (neutropenia, nausea/vomiting, and esophagitis). There were no deaths. Incidentally, one patient manifested a complete pathologic response, three a partial pathologic response, and one stable disease.

**Conclusion:**

These preliminary observations on safety suggest that this regimen can be further studied in this clinical setting.

Despite aggressive therapy with chemotherapy, radiation, and subsequent surgery, most patients with locally advanced esophageal cancer ultimately die from their disease [1]. This sobering outcome points to a clear need to begin to define cancer treatment strategies that are well tolerated and that nonetheless yield favorable clinical outcomes.

In view of this unmet need, a recent study from Siewert and others appears particularly intriguing [2]. These investigators reported on a phase I study with the novel, promising combination of pemetrexed, carboplatin, and radiation. The former is a multitargeted antifolate and preclinical and early clinical studies suggest this agent carries activity in patients with gastrointestinal malignancies, most relevantly, gastric cancer [3]. Of note, although Siewert and others found that this regimen was well tolerated, none of the esophageal cancer patients in this study was treated at the recommended maximally tolerated dose of pemetrexed 500 mg/m^2^, carboplatin AUC = 6, concurrently with radiation. Thus, this brief report provides the critically needed tolerability profile of this trimodality, neoadjuvant approach to enable investigators to further evaluate this combination in this setting.

## Methods

### Overview

The Mayo Clinic Institutional Review Board approved the study protocol before patient enrollment, and all patients provided signed informed consent.

### Patient eligibility and exclusion criteria

Salient eligibility criteria for the trial included the following: 1) age ≥ 18 years; 2) potentially resectable squamous cell cancer or adenocarcinoma of the esophagus or gastroesophageal junction (patients with celiac or supraclavicular node involvement were eligible depending on the location of the primary tumor, and patients with T1N0M0 and T2N0M0 tumors were not eligible); 3) able to receive radiation, 4) Eastern Cooperative Oncology Group performance score of 2 or better; 5) a pretreatment absolute neutrophil count of ≥1500/mL, platelet count of ≥100,000/mL, hemoglobin ≥10 g/dL, total bilirubin ≤1.5 × the upper limit of normal, aspartate transaminase ≤3 × the upper limit of normal, and a calculated creatinine clearance ≥45 mL/minute; and 6) able to discontinue non-steroidal anti-inflammatory agents, as outlined in the pemetrexed package insert.

Similarly, notable exclusion criteria included the following: 1) pregnancy or unwillingness to utilize contraception if pregnancy is a possibility; 2) illness or infection that would make the patient unable to tolerate study treatment; 3) prior radiation that would overlap anticipated radiation fields or radiation to >30% of the marrow cavity; 4) prior chemotherapy for esophageal cancer; 5) prior malignancy in the preceding 5 years with a notable risk for metastatic potential; or 6) previous reaction to pemetrexed or carboplatin.

### Pretreatment, treatment, and follow-up

All patients underwent a pretreatment history and physical examination as well as the testing needed to verify that they were meeting the eligibility criteria. Patients were also required to have completed computerized tomography of the chest and abdomen, an esophagoscopy, and an electrocardiogram.

A bronchoscopy and/or a bone scan were recommended in the event the treating oncologist had a clinical suspicion of high diagnostic yield. Endoscopic ultrasound was not mandated by the study protocol.

Patients were then monitored weekly throughout the radiation and concurrent chemotherapy administration period with a history, physical examination, and twice-a-week hemogram. Prior to the administration of chemotherapy, a chemistry profile was also required.

Tumor assessments were performed prior to surgery with the RECIST criteria [4]. Postoperatively, patients were assessed for pathologic response.

### Chemotherapy, radiation, and surgery

Chemotherapy consisted of pemetrexed 500 mg/m^2^ intravenously and carboplatin with an area under curve (AUC) of 6 intravenously on days 1 and 22. All patients were prescribed folic acid and vitamin B12 starting seven or more days prior to protocol treatment, as per the pemetrexed package insert. Chemotherapy dose reductions on day 22 were specified in the protocol and required that chemotherapy either be held, dose-reduced, or completely discontinued based on type and severity of adverse event.

Radiation was prescribed at a total dose of 5040 centigray with 180 centigray per fraction for a total of 28 fractions over 5.5 weeks. Treatment was to be held in the event of an absolute neutrophil count of <1000/mL or a platelet count of <50,000/mL.

An esophagectomy was to be performed within 4–12 weeks after completion of the concurrent chemotherapy and radiation.

### Analyses

This report describes a planned safety analysis of the first 6 consecutive patients enrolled on a phase II trial. It was decided *a priori* that if none of the 6 patients suffered a treatment-related grade 5 adverse event from the time of study initiation to 1-month post-surgery, the remaining cohort would be enrolled.

Unless otherwise specified, all adverse events are reported regardless of attribution to study treatment.

## Results

### Demographics

Demographics for these first 6 patients are shown in Table 1. Of the 6 patients 5 were male, and the median age of the cohort was 67 years (range 52–75). Five patients had an Eastern Cooperative Oncology Group Performance score of 0, and one had a score of 1. All patients had tumors of adenocarcinoma histology.

### Treatment administration

All 6 patients received full-dose pemetrexed and carboplatin during the first cycle. During the second cycle, however, adverse events prompted 3 of 6 patients to receive chemotherapy dose reductions. Two patients received approximately half the dose prescribed in the first cycle, and one received roughly 75%.

All patients completed all their intended radiation, and all underwent surgery. One patient’s surgery was delayed by one week beyond the interval specified in the protocol for medical reasons.

### Adverse events

Only 1 of the 6 patients experienced a grade 4 adverse event, which consisted of neutropenia. This patient also experienced a grade 3 depression. Of the remaining 5 patients, three experienced at least one grade 3 adverse event (neutropenia, nausea, vomiting, and esophagitis). There were no deaths, and all patients were carefully evaluated for vital status post-operatively (Table 2). All adverse events of grade 3 or worse occurred during or shortly after neoadjuvant therapy. No severe post-operative adverse events occurred.

### Response data

One patient had a complete pathological response, 3 had a partial pathological response, and one had stable disease.

## Discussion

This report describes the results of a planned interim toxicity analysis that focused on the first 6 patients enrolled on a multi-institutional trial for patients with locally advanced esophageal cancer. The combination of pemetrexed and carboplatin, as prescribed here, in conjunction with concomitant radiation and followed by surgery is well tolerated and appears to merit further study in this setting.

Although this brief report encompasses only 6 patients, the adverse event data described here are necessary to permit the further study of this regimen in patients with locally advanced esophageal cancer. Previous studies have demonstrated that some regimens offer limited survival benefits while providing a substantial adverse event profile, including hospitalization rates that exceed 50%, grade 3 or 4 adverse event rates that go beyond 70%, and treatment-related death rates that occur in over 5% of patients [5]. Therefore, reporting preliminary safety data for a promising regimen, as summarized in this brief report, provides the necessary groundwork for further study of such a regimen, especially when the requisite phase I data are lacking.

Thus, based on the data presented here, the North Central Cancer Treatment Group is proceeding with a multi-institutional phase II trial that tests the foregoing regimen in patients with locally advanced esophageal cancer.

## Figures and Tables

**Table 1:** Patient demographics (n **=** 6)*.

Age in years median (range)	67 (52–75)
Male: Female	5:1
Eastern Cooperative Oncology Group performance score	
0	5
1	1

* Numbers denote number of patients unless otherwise specified.

**Table 2:** Grade 3 or worse maximal adverse events (N **=** 6)*.

Adverse event	Number of patients
neutropenia	2
depression	1
nausea/vomiting	1
esophagitis	1

* Other events such as anemia, thrombocytopenia, neuropathy, diarrhea, and dehydration were solicited but not detected.
